# Draft Genome Sequence of the Polychlorinated Biphenyl-Degrading Bacterium *Comamonas testosteroni* KF712 (NBRC 110673)

**DOI:** 10.1128/genomeA.01214-15

**Published:** 2015-10-15

**Authors:** Jun Hirose, Atsushi Yamazoe, Akira Hosoyama, Nobutada Kimura, Hikaru Suenaga, Takahito Watanabe, Hidehiko Fujihara, Taiki Futagami, Masatoshi Goto, Kensuke Furukawa

**Affiliations:** a Department of Applied Chemistry, Faculty of Engineering, University of Miyazaki, Miyazaki, Japan; b Biological Resource Center, National Institute of Technology and Evaluation (NITE), Tokyo, Japan; c Bioproduction Research Institute, National Institute of Advanced Industrial Science and Technology (AIST), Tsukuba, Japan; d Research Institute for Sustainable Humanosphere, Kyoto University, Uji, Kyoto, Japan; e Department of Food and Nutrition, Beppu University, Beppu, Japan; f Education and Research Center for Fermentation Studies, Faculty of Agriculture, Kagoshima University, Kagoshima, Japan; g Laboratory of Future Creation Microbiology, Faculty of Agriculture, Kyushu University, Fukuoka, Japan

## Abstract

We present a 5.89-Mb draft genome sequence of *Comamonas testosteroni* KF712 (NBRC 110673), a polychlorinated biphenyl degrader. The genome sequence clarified that KF712 harbors the gene clusters coding for the catabolism of biphenyl and at least seven other aromatic compounds.

## GENOME ANNOUNCEMENT

For some time now, polychlorinated biphenyls (PCBs) have been recognized as serious environmental contaminants on a global scale. Certain PCBs are cometabolized by biphenyl-utilizing bacteria. We have isolated 14 PCB-degrading bacterial strains (KF strains), including *Comamonas testosteroni* KF712 (formerly known as *Pseudomonas* sp. strain KF712), from the soil near a biphenyl manufacturing plant in Kitakyushu, Japan, by enrichment culture with biphenyl (1). Degradation of biphenyl and biphenyl-related compounds by *C. testosteroni* KF712 has been investigated using transposon mutants (2). Several other strains of *C. testosteroni* are known to mineralize complex xenobiotic compounds, such as testosterone (3) and 4-chloronitrobenzene (4), but do not assimilate carbohydrates because they lack a part of the genes involved in glycolysis and the pentose phosphate pathway (5).

The draft genome sequence of KF712 was determined by the National Institute of Technology and Evaluation (NITE) using a combination of shotgun sequencing with 454 GS FLX+ system (Roche) and paired-end sequencing using the HiSeq 1000 system (Illumina). The reads obtained by the two systems were assembled using the Newbler software package version 2.6 (Roche). The assembled genome is composed of 97 contigs (>601 bp) totaling 5,890,323 bases, with a G+C content of 61.3%. The *N*_50_ contig size and the largest contig size are 131,478 bp and 377,061 bp, respectively.

The draft genome sequence of the KF712 strain annotated using RAST version 2.0 (6) contains 5,563 predicted coding DNA sequences (CDSs), three rRNAs (5S, 16S, and 23S), and 57 tRNA sequences. The coding sequences were classified into 465 subsystems, with the most abundant systems being those involved in the metabolism of amino acid derivatives (*n* = 481 CDSs) and carbohydrates (*n* = 348); cofactors, vitamins, prosthetic groups, and pigments (*n* = 342); fatty acids, lipids, and isoprenoids (*n* = 229); respiration (*n* = 214); membrane transport (*n* = 212); and protein metabolism (*n* = 210). Comparison of genome sequences available in the RAST data sets revealed that *Comamonas testosteroni* KF-1 (7) is the closest neighbor of the KF712 strain with a score of 527, followed by *C. testosteroni* CNB-2 with a score of 512 (5).

The *bph* gene cluster (*bphEGFA1A2A3BCDA4*) involved in biphenyl/PCB degradation and a portion of the *trb* gene cluster (*trbBCDEJ*) responsible for conjugative transfer were found in a single contig. They were almost identical to the components of the biphenyl catabolic transposon Tn*4371* from *Cupriavidus oxalaticus* A5 (8) and those of ICE_KKS102_*4677* from *Acidovorax* sp. strain KKS102 (9). In addition, the genes clusters involved in the degradation of at least seven other aromatic compounds such as benzoate, gentisate, phenol, phenylacetate, terephthalate, isophthalate, and vanillate were found, which accounted for the ability of the bacterium to grow on these compounds. The information obtained on the genome sequence of *C. testosteroni* KF712 offers an opportunity to understand the unique carbon catabolism and adaptive measures used by bacteria in environments polluted by aromatic compounds.

### Nucleotide sequence accession numbers.

The draft genome sequence of *C. testosteroni* KF712 has been deposited in DDBJ/EMBL/GenBank under accession numbers BBQP01000001 to BBQP01000097.
